# DArTseq genotyping facilitates identification of *Aegilops biuncialis* chromatin introgressed into bread wheat Mv9kr1

**DOI:** 10.1007/s11103-024-01520-2

**Published:** 2024-11-07

**Authors:** Eszter Gaál, András Farkas, Edina Türkösi, Klaudia Kruppa, Éva Szakács, Kitti Szőke-Pázsi, Péter Kovács, Balázs Kalapos, Éva Darkó, Mahmoud Said, Adam Lampar, László Ivanizs, Miroslav Valárik, Jaroslav Doležel, István Molnár

**Affiliations:** 1 Department of Biological Resources, Centre for Agricultural Research, Hungarian Research Network, Martonvásár, 2462 Hungary; 2 Institute of Experimental Botany of the Czech Academy of Sciences, Centre of Plant Structural and Functional Genomics, Olomouc, 77900 Czech Republic; 3 Agricultural Research Centre, Field Crops Research Institute, 9 Gamma Street, Giza, 12619 Egypt

**Keywords:** *Aegilops biuncialis*, Chromosome addition lines, DArTseq analysis, Thousand-grain weight, Wheat-*Aegilops* introgressions

## Abstract

Wild wheat relative *Aegilops biuncialis* offers valuable traits for crop improvement through interspecific hybridization. However, gene transfer from *Aegilops* has been hampered by difficulties in detecting introgressed U^b^- and M^b^-genome chromatin in the wheat background at high resolution. The present study applied DArTseq technology to genotype two backcrossed populations (BC382, BC642) derived from crosses of wheat line Mv9kr1 with *Ae. biuncialis* accession, MvGB382 (early flowering and drought-tolerant) and MvGB642 (leaf rust-resistant). A total of 11,952 *Aegilops*-specific Silico-DArT markers and 8,998 wheat-specific markers were identified. Of these, 7,686 markers were assigned to U^b^-genome chromosomes and 4,266 to M^b^-genome chromosomes and were ordered using chromosome scale reference assemblies of hexaploid wheat and *Ae. umbellulata*. U^b^-genome chromatin was detected in 5.7% of BC382 and 22.7% of BC642 lines, while 88.5% of BC382 and 84% of BC642 lines contained M^b^-genome chromatin, predominantly the chromosomes 4M^b^ and 5M^b^. The presence of alien chromatin was confirmed by microscopic analysis of mitotic metaphase cells using GISH and FISH, which allowed precise determination of the size and position of the introgression events. New Mv9kr1-*Ae. biuncialis* MvGB382 4M^b^ and 5M^b^ disomic addition lines together with a 5DS.5DL-5M^b^L recombination were identified. A possible effect of the 5M^b^L distal region on seed length has also been observed. Moreover, previously developed Mv9kr1-MvGB642 introgression lines were more precisely characterized. The newly developed cytogenetic stocks represent valuable genetic resources for wheat improvement, highlighting the importance of utilizing diverse genetic materials to enhance wheat breeding strategies.

**Supplementary Information:**

The online version contains supplementary material available at 10.1007/s11103-024-01520-2.

## Key message

DArTseq genotyping supported by chromosome-scale assemblies of wheat and *Aegilops* genomes facilitates the selection of wheat‑*Aegilops biuncialis* addition, substitution and translocation lines.

**Supplementary Information:**

The online version contains supplementary material available at 10.1007/s11103-024-01520-2.

## Introduction

The limited genetic diversity of hexaploid bread wheat (*Triticum aestivum* L.; 2n = 6x = 42; AABBDD) poses a significant challenge for breeders to select optimal combinations of gene variants determining adaptability, tolerance to biotic and abiotic stresses and grain quality traits under changing environment. Wild relatives of wheat are huge reservoirs of useful traits and alleles that can be utilized in wheat improvement through interspecific or intergeneric hybridization. The genus *Aegilops* comprises 23 species, including 11 diploid, 10 allo-tetraploid and 2 allo-hexaploid. Seven different genomes (C, D, M, N, S, T and U) were identified in the diploids, and most of them can also be found in the allo-tetraploid and allo-hexaploid species (van Slageren 1994). Genetic diversity of the genus makes the *Aegilops* species a large and attractive reservoir of useful genes for wheat improvement.

Allo-tetraploid *Aegilops biuncialis* Vis. (2n = 4x = 28; U^b^U^b^M^b^M^b^) belongs to the tertiary gene pool of bread wheat. The species is native to the Mediterranean and Western Asiatic regions, characterised by dry summer seasons with high temperatures and a wide range of annual rainfall (225–1250 mm). Adaptation to such diverse geographical conditions resulted in a great genetic variability of the species (Ivanizs et al. 2019). Some accessions of *Ae. biuncialis* are resistant to barley yellow dwarf virus (Makkouk et al. 1994) and fungal diseases such as powdery mildew (Li et al. 2019) and yellow-, leaf- or stem rusts (Damania and Pecetti 1990; Dimov et al. 1993; Kwiatek et al. 2012; Olivera et al. 2018). Other accessions have been found useful sources of genes contributing to cold-, salt- or drought tolerance (Colmer et al. 2006; Darkó et al. 2020; Dulai et al. 2014; Molnár et al. 2004; Rekika et al. 1997), nutritional quality including high content of micro- and macronutrients such as iron and zinc (Farkas et al. 2014), and also high protein and dietary fibre content of the flour (Rakszegi et al. 2017, 2019).

Useful traits of *Ae. biuncialis* can be utilized in wheat breeding programs through interspecific hybridization resulting in wheat-*Ae. biuncialis* introgression lines. Tan et al. (2009) produced a partial wheat-*Ae. biuncialis* amphiploid, Zhou et al. (2014) developed an 1U^b^ wheat-*Ae. biuncialis* addition line, while Song et al. (2020) selected a 5M^b^ disomic addition line. Using gene bank accession MvGB642 originating from a humid habitat in Syria, a Hungarian research group developed several winter wheat (‘Mv9kr1’) - *Ae. biuncialis* cytogenetic stocks, such as chromosome addition lines 2M^b^, 3M^b^, 7M^b^, 3U^b^, 4U^b^ and 5U^b^ (Schneider et al. 2005), chromosome substitution lines 3M^b^(4B), 4M^b^(4D) and 5M^b^(5D), and introgressions including Robertsonian translocations 3M^b^∙4BS and 1U^b^S∙2BL (Farkas et al. 2014). Several terminal translocations involving U^b^ or M^b^ genomic fragments were also developed, but the precise identification of the introgressed *Aegilops* chromosome fragments has been hampered by the lack of diagnostic cytogenetic- and molecular markers (Farkas et al. 2014).

Until now, *Ae. biuncialis* chromosome fragments have been introgressed into wheat from only a few accessions, despite the fact that a large number of *Ae. biuncialis* accessions are maintained in gene banks around the world (Adhikari et al. 2023; Monneveux et al. 2000). To utilize the untapped genetic diversity of *Ae. biuncialis* in breeding programs, more gene bank accessions should be included in gene introgression programs. *Ae. biuncialis* accession MvGB382 originates from a habitat with low annual rainfall (550 mm/year) and flowers more than a week earlier compared to MvGB642 (Ivanizs et al. 2019), indicating the presence of an escape molecular mechanism avoiding harmful effects of heat- and drought stress as frequently observed in early summer in Central Europe (Zaharieva et al. 2001). MvGB382 has also been reported to be more tolerant to drought and salt (Molnár et al. 2004) and to have better grain quality, such as high dietary fibre or micronutrient content, than the previously used accession MvGB642 (Farkas et al. 2014; Rakszegi et al. 2017, 2019). With the aim to transfer useful gene variants from the MvGB382 accession into wheat, wheat line Mv9kr1 was crossed with *Ae. biuncialis* MvGB382 and backcrossed (BC) to produce BC_2_, BC_3_, and BC_3_F_2_ generations. However, the success in the detection of gene introgression from *Ae. biuncialis* into wheat depends on the ability to screen large populations for the presence of alien chromatin, and it is also necessary to identify particular wheat and alien chromosomes involved in the chromosome rearrangements.

Some of the most popular techniques for identifying alien chromatin in the wheat background are molecular cytogenetic methods. Genomic in situ hybridization (GISH), using labelled total genomic DNA of the introgressed species as a probe, allows visualization of the alien chromosomes and chromosome segments (Schwarzacher et al. 1989). *Ae. biuncialis* U^b^ and M^b^ chromosome segments were also detected simultaneously using multicolour GISH with labelled genomic DNA of *Ae. umbellulata* (2n = 2*x* = 14; UU) and *Ae. comosa* (2n = 2*x* = 14; MM) by Molnár et al. (2009) in *T. aestivum* x *Ae. biuncialis* amphiploids. Karyotype of *Ae. biuncialis* produced by fluorescence in situ hybridization (FISH) using DNA repeat probes (Afa-family, pSc119.2 and 45S rDNA) allowed to identify *Aegilops* chromosomes and chromosome segments in the wheat genetic background (Farkas et al. 2014; Kwiatek et al. 2013; Schneider et al. 2005). While molecular cytogenetic methods are powerful techniques, they are less efficient for screening large pre-breeding populations or identifying cryptic introgressions (Kuraparthy et al. 2009).

Due to their high throughput, the application of molecular markers offers an alternative for the selection of wheat-alien introgressions (Rey et al. 2015). The first generation of molecular markers, such as restriction fragment length polymorphism (RFLP) and random amplified polymorphic DNA (RAPD) did not require prior sequence information. Similar to most of hybridization-based markers, their application was time-consuming, labour-intensive, and expensive. Nevertheless, these markers allowed the identification wheat-alien chromosome and chromosome-arm addition and substitution lines (King et al. 1993; Qi et al. 1997).

Simple sequence repeat (SSR) and amplified fragment length polymorphism (AFLP) markers along with their variants belong to the second generation of markers and are mostly medium-throughput and PCR-based. The third generation of molecular markers such as expressed sequence tags (ESTs) and single nucleotide polymorphisms (SNPs) are high-throughput sequence-based markers (Singh and Singh 2015). While several molecular markers have been tested in U- and M-genome species of *Aegilops*, such as RFLP, RAPD, or gene-specific PCR-based Landmark Unique Gene (PLUG) or Conserved Ortholog Set (COS) markers (Gong et al. 2017; Molnár et al. 2013), only some were specific for *Ae. biuncialis*. Moreover, the exact location and the order of markers along the chromosomes could not be determined, limiting the use of these markers to detect alien introgressions in wheat pre-breeding populations.

The advent of next-generation sequencing technologies allowed high-resolution characterization of the large and complex genome of hexaploid wheat (IWGSC 2014, 2018). In case of the diploid progenitors of *Ae. biuncialis*, a high-quality chromosome-scale reference sequences have only recently become available for the U genome (Abrouk et al. 2023), but only low-quality draft assemblies are available for the M genome chromosomes (Said et al. 2021).

Based on the reduced representation sequencing approach, new marker technologies have been developed to characterize the genome at hundreds of thousands of data points in crop wild relatives (Jing et al. 2009; Olivera et al. 2013). Diversity Arrays Technology (DArT) was originally a hybridization-based microarray platform (Wenzl et al. 2004), which used genome complexity reduction based on double digestion of genomic DNA with a methylation-sensitive restriction endonuclease (such as *PstI*) along with a frequently cutting enzyme (like *TaqI*). A combination of the DArT genome complexity reduction method and genotyping-by-sequencing approach led to the development of the DArTseq technology (Elshire et al. 2011). The technology results in two types of markers, namely Silico-DArT and SNP-DArT markers. The dominant Silico-DArT markers indicate the presence/absence variation of the genomic fragment, while the codominant SNP-DArT markers represent the nucleotide polymorphism within the genomic fragment.

Since the DArTseq technology does not require prior sequence information, it has been widely applied for genotyping, genetic diversity analysis, and genome-wide association studies (GWAS) in wild relatives of wheat such as *Triticum monococcum* (Jing et al. 2009), *Ae. tauschii* (Kumar et al. 2015) and *Ae. sharonensis* (Olivera et al. 2013). DArTseq markers were also used to study the population structure of an *Ae. biuncialis* diversity collection containing 86 accessions with diverse eco-geographic origins (Ivanizs et al. 2019). In parallel to the present study, used DArTseq to genotype an F_2_ population developed from a cross of *Ae. biuncialis* MvGB382 x MvGB642 to construct a high-resolution genetic map. Using the mapped markers, the authors investigated wheat-*Aegilops* syntenic relationship, highlighting a well-preserved collinearity of the M^b^ genome chromosomes with those of wheat and the rearranged structure of the U^b^ chromosomes.

Motivated by the need for a high-resolution and high-throughput screening system in wheat alien introgression breeding, we used DArTseq technology to detect *Ae. biuncialis* chromatin in the wheat genetic background. We applied the wheat-*Ae. biuncialis* synteny information to order the markers identified on the U^b^ and M^b^ chromosomes using the results from the wheat and *Aegilops* parents, the diploid progenitors *Ae. umbellulata* and *Ae. comosa*, respectively, and the wheat-*Ae. geniculata* addition lines. Chromosome-specific markers were used to study two wheat x *Ae. biuncialis* BC_3_ pre-breeding populations containing genetic variation from the wild accessions MvGB382 and MvGB642. The combined use of genomic- and DNA repeat probes for in situ hybridization and DArTseq allowed to select new wheat-*Ae. biuncialis* MvGB382 chromosome addition and translocation lines and the identification of chromosomal segments involved in previously detected wheat-*Ae. biuncialis* MvGB642 translocations.

## Materials and methods

### Plant material

Bread wheat x *Ae. biuncialis* BC_3_ pre-breeding populations were developed as described previously by Farkas et al. (2023). Briefly, the winter wheat genotype Mv9kr1 was crossed with *Ae. biuncialis* accessions MvGB382 and MvGB642. F_1_ hybrids were treated with colchicine to produce amphiploids which were backcrossed with the parental wheat genotype Mv9kr1 to produce the Mv9kr1/MvGB642 backcrossed (BC_3_) population (hereafter abbreviated as BC642). The production of Mv9kr1 x *Ae. biuncialis* MvGB382 BC_3_ population (BC382) followed the same procedure as BC642. 35 BC382 and 44 BC642 genotypes were screened for the presence of *Ae. biuncialis* chromatin by DArTseq and in situ hybridization. For DArTseq genotyping, the BC382 and BC642 populations were investigated together with the parental wheat (Mv9Kr1) and four wheat genotypes as control (Chinese Spring, Hombár#1, Hombár#2, Ménrót), the parental *Ae. biuncialis* genotypes, diploid progenitors of the U and M genomes, namely *Ae. umbellulata* accession AE740/03 and *Ae. comosa* accession MvGB1039. Wheat (‘Chinese Spring’)/*Ae. geniculata* (2n = 4x = 28; U^g^U^g^M^g^M^g^) (Friebe et al. 1999) chromosome additions 1M^g^-7M^g^, Chinese Spring/*Ae. umbellulata* additions 1U, 2U, 4U-7U (kindly provided by Dr. Steve Reader, John Innes Centre, Norwich, UK) and Mv9kr1/*Ae. biuncialis* MvGB642 addition line 3U^b^ (Schneider et al. 2005), representing the whole set of U^b^ and M^b^ genome chromosomes, were also DArTseq genotyped as a control. The plant material used in the present study is summarized in Supplementary Table 1.

### Molecular cytogenetic analysis

Mitotic metaphase chromosome preparations, probe labelling and in situ hybridization experiments (GISH and FISH) on BC382 and BC642 populations were done as described in Farkas et al. (2023). Briefly, microscopic slides with mitotic chromosome spreads were prepared from synchronized root tip meristematic cells. Total genomic DNA was isolated from *Ae. umbellulata* AE740/03 and *Ae. comosa* MvGB1039, labelled with biotin-16-dUTP (Roche, Mannheim, Germany) or digoxigenin-11-dUTP (Roche), and used as the U- or M-genomic probe, respectively. Unlabelled genomic DNA from wheat Mv9kr1 was used to block unspecific hybridization.

The pSc119.2 and Afa-family sequences were amplified and labelled with biotin-16-dUTP (Roche) and digoxigenin-11-dUTP (Roche), respectively, using PCR (Contento et al. 2005; Nagaki et al. 1995). The pTa71 clone specific for the 45 S rDNA (Gerlach and Bedbrook 1979) was separately labelled with biotin-16-dUTP and digoxigenin-11-dUTP and then applied as a 0.5:0.5 v/v mixture. Digoxigenin- and biotin-labelled probes were detected using anti-digoxigenin-rhodamine Fab fragments (Roche) and Alexa Fluor-488 streptavidin conjugate (Invitrogen, Life Technologies, Carlsbad, USA), respectively.

Sequential in situ hybridizations with genomic and DNA repeat probes were performed as described by Molnár et al. (2009). Briefly, hybridization mix (25 µl per slide) contained 70 ng of each labelled probe. In case of GISH, 3500 ng of wheat genomic DNA was included in the hybridization mix as blocking DNA. The hybridization was carried out overnight at 42^°^C under humid conditions.

Chromosomes were counterstained with 2 µg/ml DAPI (4’,6-diamidino-2-phenylindole, Amersham) and fluorescence signals were visualized using a Zeiss AxioImager M2 epifluorescence microscope equipped with filter sets for DAPI, FITC, and Rhodamine signals. Images were captured with a Zeiss AxioCam MRmCCD camera and processed with Zeiss AxioVision 4.8.2 software. After visualization of GISH signals, the slides were re-probed with probes for Afa-family, pSc119.2, and pTa71 DNA repeats using the washing steps and hybridization solution described by Molnár et al. (2009).

### Morphological characterization

The plant material developed in this study was grown under glasshouse conditions as described by Rakszegi et al. (2017). Seed morphology was analysed with MARViN Digital Seed Analyser System. The results from the glasshouse experiment represent the means ± standard deviation for various measurements per genotype, such as spike length, number of spikelets, number of seeds, yield, thousand-grain weight (TKW), seed length and seed width (see Tables 1 and 2). Differences between the pre-breeding lines and the parental wheat line Mv9Kr1 were determined using Student’s t-test for paired data at the *P* = 0.05 significance level.

**Table 1:** Morphological traits of Mv9kr1, wheat-*Ae. biuncialis* 5M^b^ disomic addition (382), wheat-*Ae. biuncialis* 4M^b^ disomic addition (382), substitution 5M^b^(5D) and translocation lines grown in the glasshouse (2023, Martonvásár). Values are the means ± standard deviations

Genotype	No. of measurements	Spike length (cm)	No. of spikelets	No. of seeds	Fertility
**Mv9kr1**	10	9.00 ± 1.07	17.90 ± 1.64	47.30 ± 6.83	2.64 ± 0.26
**5M** ^**b**^ **add 382**	5	11.20* ± 1.03	17.80 ± 2.48	25.80* ± 8.84	1.42* ± 0.31
**4M** ^**b**^ **add 382**	5	7.30* ± 0.68	21.00* ± 1.26	27.40* ± 5.50	1.32* ± 0.34
**T5DS.5DL-5M** ^**b**^ **L #1 (382)**	6	11.50* ± 1.29	24.67* ± 1.80	47.50 ± 18.99	1.88* ± 0.65
**T5DS.5DL-5M** ^**b**^ **L #2 (642)**	4	9.13 ± 1.14	17.75 ± 2.05	16.50* ± 6.87	0.90* ± 0.28
**5M** ^**b**^ **(5D) 642**	10	12.05* ± 0.57	21.90* ± 0.83	58.00* ± 3.46	2.65 ± 0.12

*Significantly different from the value of parental wheat Mv9kr1 at *p* = 0.05

**Table 2:** Grain morphological traits of Mv9kr1, *Ae. biuncialis* MvGB382 and MvGB642, wheat-*Ae. biuncialis* 4M^b^ disomic addition (382), wheat-*Ae. biuncialis* 5M^b^ disomic addition (382), substitution 5M^b^(5D) and translocation lines grown in the glasshouse (2023, Martonvásár). Values are the means ± standard deviations

Genotype	Number of measurements	TKW (g)	Seed	
			Width (mm)	Length (mm)
***T. aestivum*** **‘Mv9kr1’**	10	40.22 ± 3.33	3.48 ± 0.13	6.24 ± 0.11
***Ae. biuncialis*** **MvGB382**	2	23.02*^,#^ ± 0.18	2.90* ± 0.02	6.99* ± 0.26
***Ae. biuncialis*** **MvGB642**	2	16.57* ± 0.30	2.98* ± 0.03	6.33 ± 0.07
**5M** ^**b**^ **add 382**	5	52.53* ± 2.75	3.76* ± 0.25	7.79* ± 0.29
**4M** ^**b**^ **add 382**	2	22.71 ± 2.81	3.01* ± 0.03	5.82* ± 0.04
**T5DS·5DL-5M** ^**b**^ **L #1 (382)**	3	46.59 ± 7.79	3.26* ± 0.12	7.73* ± 0.19
**T5DS·5DL-5M** ^**b**^ **L #2 (642)**	2	30.08* ± 0.00	3.03* ± 0.01	5.72* ± 0.02
**5M** ^**b**^ **(5D) 642**	3	31.77* ± 1.06	3.23 ± 0.02	6.20 ± 0.06

*Significantly different from the value of parental wheat Mv9kr1 at *p* = 0.05

#Significantly different from the value of *Ae. biuncialis* 642 at *p* = 0.05.

### DArTseq genotyping

Genomic DNA was extracted from fresh young leaves of the BC_3_ plants and control genotypes (wheat and *Aegilops* parental lines, diploid genome progenitors, and wheat/*Aegilops* chromosome additions) summarized in Supplementary Table 1, using a BioSprint DNA Plant Kit (Qiagen, Hilden, Germany) on a BioSprint 96 Workstation (Qiagen) according to the manufacturer’s instructions. The DNA samples were genotyped using the Diversity Arrays Technologies Pty. Ltd., Australia (http://www.diversityarrays.com) using the ‘wheat DArTseq™ 1.0’ platform.

The resulting Silico-DArT markers were scored as binary data (1 or 0) reflecting the presence or absence of a marker in the genomic representation of each sample. Based on the presence/absence polymorphism between wheat and *Ae. biuncialis* parental lines, Silico-DArT markers were split into wheat- and *Aegilops*-specific groups, while the markers showing low quality genotype call on wheat or *Aegilops* parents (’-‘) were excluded from the analysis. *Aegilops*-specific markers were further split into U- and M-genome specific groups according to the genotype data (presence/absence polymorphism) on diploid progenitors, *Ae. umbellulata* and *Ae. comosa*, respectively. Non-polymorphic markers between wheat and *Ae. biuncialis* genotypes and between the U and M genomes were excluded from the analysis.

In order to determine chromosomal location of the selected markers in wheat, the marker sequences were used as queries for BLASTn searches against the reference pseudomolecules of the A, B and D sub-genomes of hexaploid wheat; Ensembl Plants, release-46 (IWGSC 2018). The start position of each wheat-specific Silico-DArT sequence was determined based on the best hit obtained using the BLASTn package of the Blast Command Line Application 2.9.0 (http://ftp.ncbi.nlm.nih.gov/) with the following parameters: -task ‘BLASTn’; -evalue 1e^− 5^; -max_target_seqs 2; -max_hsps 1. The markers assigned to wheat chromosomes were ordered by the start position of the best hits (summarized in Supplementary Data S1).

Wheat-*Ae. biuncialis* syntenic relationships, obtained by genetic mapping were used to determine deviations of *biuncialis* chromosomal structure from the bread wheat. Because the *Ae. biuncialis* genetic map showed that the M^b^ chromosomes are collinear with the corresponding chromosomes of the wheat D sub-genome, the Silico-DArT markers assigned to the M genome in the present study were also BLAST compared against the wheat A, B and D sub-genomes. The M genome markers with significant hits on the D sub-genome (the hits were excluded if the length of the alignment was ≤ 50% of the marker sequence and E-value ≥ 10^− 6^) were grouped by their chromosomal location and ordered by the position of the best hits on the D chromosomes (summarized in Supplementary Data S2).

Finally, the Silico-DArT markers specific for the U genome were BLAST compared against the chromosome-scale reference sequence assemblies of *Ae. umbellulata* TA1851 (Abrouk et al. 2023) and their order on chromosomes was determined by the best hits (summarized in Supplementary Data S3).

Chromosomal location of each marker was also validated by the genotype data on the set of wheat/*Aegilops* addition lines representing each of the U^b^ and M^b^ genome chromosomes in the wheat background (summarized in Supplementary Table 1.).

### Coverage estimation

For the coverage analysis, the chromosomes of the wheat and *Aegilops* species were divided into non-overlapping windows of 1 Mb. For each window, the number of mapped Silico-DArT markers was counted. The distribution of the marker density along the chromosomes was visualized on heat maps using MeV 4.9.0. (Multiple Experiment Viewer) software. Subsequently, the counts were normalized by dividing them by the number of markers assigned to the given chromosome. To identify alien chromatin in a BC382 and BC642 lines, the normalized counts of each chromosome of a BC plant were divided by the counts of the parental *Ae. biuncialis* lines.

To determine the wheat chromosome involved in the translocations, the counts of mapped wheat-specific Silico-DArT markers were used for the comparison of parental wheat (Mv9kr1) and BC lines in the same way as it was described for the detection of *Aegilops* chromatin.

## Results

### Assigning DArT markers to Aegilops chromosomes

We used DArTseq technology to identify *Ae. biuncialis* introgressions in hexaploid wheat Mv9kr1 BC_3_ populations. A total of 239,770 Silico-DArT markers was generated. Using the presence/absence polymorphism between the wheat genotypes and the U- and M-genomes of diploid and allotetraploid *Aegilops* accessions, we selected 11,952 markers specific for the *Aegilops* species, while 9,002 markers were specific for wheat (Table 3). The *Aegilops*-specific markers were further split into 7,686 and 4,266 markers specific for the U and M genomes using the information from progenitors *Ae. umbellulata* and *Ae. comosa*, respectively.

**Table 3:** The number of Silico-DArT markers identified on the sub-genomes of *Ae. biuncialis* and *T. aestivum*

Chr. group	Aegilops sub-genomes			T. aestivum sub-genomes		
	U	M		A	B	D
1	714	576		397	300	431
2	1261	766		223	387	463
3	851	628		497	467	423
4	1074	556		401	388	374
5	1291	699		533	398	630
6	1175	436		310	413	423
7	1320	605		486	447	607
Total	7686	4266		2847	2800	3351

The U genome-specific markers were BLAST compared against the TA1851 reference genome of *Ae. umbellulata* (Abrouk et al. 2023) to determine their positions on individual chromosomes. The lowest number of markers was assigned to 1U (714 markers), while the highest number of markers was identified on 7U (1,320 markers) (Table 3, Supplementary Data S3). Considering that the M^b^-genome chromosomes of *Ae. biuncialis* showed preserved macro synteny with the D sub-genome of wheat according to a recent MvGB382 x MvGB642 segregating genetic map, we used trimmed sequences of M^b^ genome-specific markers for a BLASTn comparison against the reference sequence assembly of the wheat D sub-genome (IWGSC 2018). Using the best hits, the lowest number of markers (436) was identified and ordered for chromosome 6 M, while the highest number of markers (766) was found for 2M (Table 3, Supplementary Data S2).

We also mapped 2,847, 2,800, and 3,351 wheat-specific markers to the wheat A, B, and D sub-genomes, respectively, and ordered the markers along the chromosomes using their positions on the cv. Chinese Spring reference genome pseudomolecules (IWGSC 2018) (Table 3. Supplementary Data S1). Finally, the chromosomal positions of the markers were validated using the hexaploid wheat/*Aegilops* chromosome addition lines, where the markers indicated the presence of *Aegilops* chromosomes in the wheat genetic background.

**Fig. 1:**
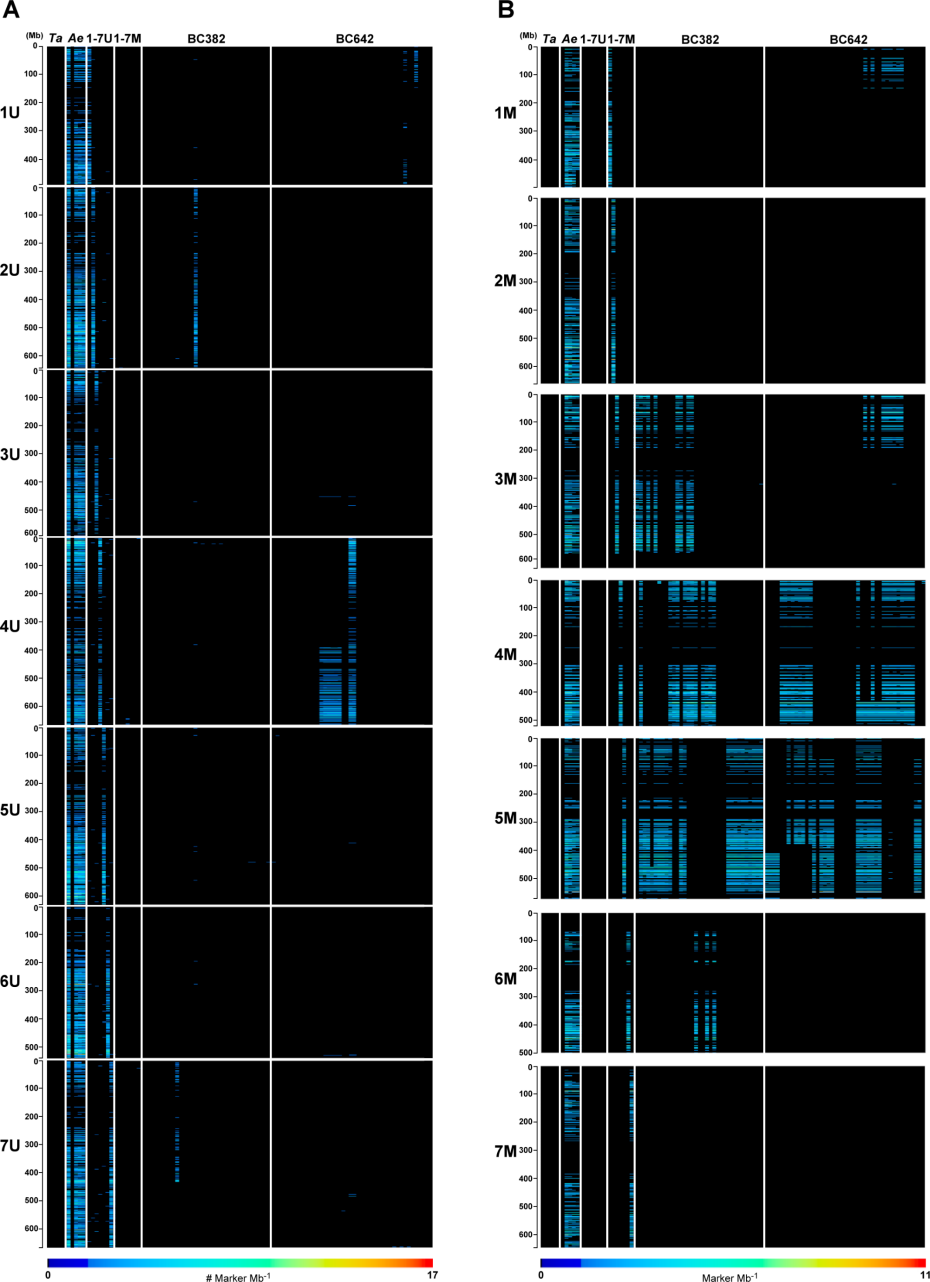
Identification of the introgressed U^b^- (**A**) and M^b^-sub-genome (**B**) chromosomes in the wheat Mv9kr1/*Ae. biuncialis* BC_3_ populations (BC642, BC382). The density of *Aegilops*-specific Silico-DArT markers (expressed as the number of markers per Mb) in seven homologous groups is shown as heatmaps. The genotypes of wheat (*Ta*: Mv9kr1, Chinese Spring, Mv Hombár#1, Mv Hombár#2, Mv Ménrót) and *Aegilops* accessions (*Ae*: *Ae. umbellulata* AE740/03, *Ae. comosa* MvGB1039, *Ae. biuncialis* MvGB382, *Ae. biuncialis* MvGB642 and Mv9kr1/*Ae. biu*. MvGB642 amfiploid), wheat/*Aegilops* addition lines representing the chromosomes of U- (1-7U) and M-genomes (1-7 M) in wheat, and the wheat/*Ae. biuncialis* BC_3_ populations (BC382: 35 lines, BC642: 44 lines) are ordered horizontally

To identify candidate introgressed regions in the BC382 and BC642 populations, the marker density and distribution on each chromosome of the *Aegilops* and wheat sub-genomes were visualized on heat maps (Fig. 1, Supplementary Fig. 1). Marker density was higher at telomeric regions of each chromosome, while it was lower at the centromeric-pericentromeric regions, irrespective of the sub-genome. In case of wheat, a relatively low number of markers was generated on chromosome 2 A and on the short arm of 1B (Supplementary Fig. 1).

**Fig. 2:**
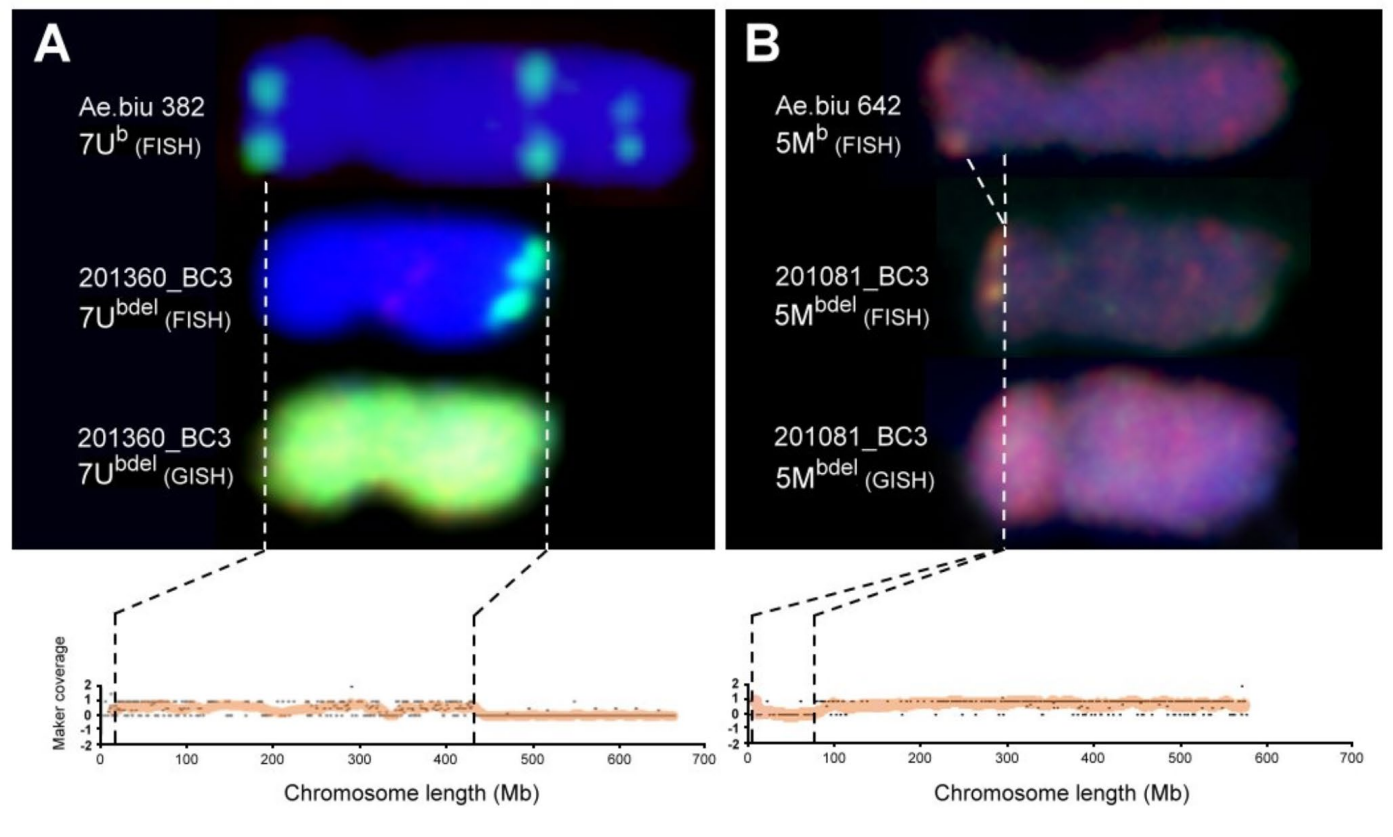
Deletions on chromosomes 7U^b^ (**A**) and 5M^b^ (**B**) in the BC382 and BC642 populations, respectively. Fluorescence in situ hybridisation (FISH) with probes for Afa-family (red), pSc119.2 (green) and 45S rDNA (yellow) DNA repeats on wild-type chromosomes from the parental *Ae. biuncialis* lines (MvGB382, MvGB642) and chromosome deletions from the BC_3_ lines (201360_BC3, 201081_BC3) together with GISH using U- (green) and M-genomic (red) probes show differences between wild type and chromosome deletions. The coverage of *Aegilops*-specific Silico-DArT markers on the corresponding chromosomes indicate the deleted regions where the expected value of marker coverage is ‘0’, while the *Aegilops* chromosome region present in the BC_3_ line is characterized by a coverage value of ‘1’

Markers specific for *Aegilops* showed a significant difference in the presence of U^b^ and M^b^ chromatin in both BC_3_ populations. Out of the 35 BC382 genotypes, two lines (5.7%) contained U^b^-genome chromatin, one (201373_BC_3_) carrying the entire chromosome 2U^b^ and one (201360_BC_3_) a deletion 7U^b^, respectively (Supplementary Data S3). Of the 44 BC642 genotypes, ten (22.7%) carried chromatin from two U^b^ chromosomes. Entire chromosome 1U^b^ was detected in line 201170_BC_3_ and the 1U^b^S chromosomal arm in line 201179_BC_3_. Eight lines contained chromatin from chromosome 4U^b^, two lines carried whole 4U^b^, while six lines contained 4U^b^L (Fig. 2., Supplementary Table 2.).

M^b^-genome chromatin was detected in 31 lines (88.5%) of the BC382 population. Chromosomes 6M^b^ (in 3 lines) and 3M^b^ (in 9 lines) were relatively rare, while chromosome 4M^b^ (in 13 lines) and 5M^b^ (in 21 lines) were quite abundant. A similar tendency was observed in BC642 population as M^b^-genome chromatin was present in 37 lines (84%). Similar to the BC382 population, chromosomes 4M^b^ (in 26 lines) and 5M^b^ (in 19 lines) were also frequently present, while the chromosomes 1M^b^ and 3M^b^ (both of them in 8 genotypes) were relatively rare (Fig. 2., Supplementary Table 2.).

Although deletions were quite abundant in wheat sub-genomes, such as a ~ 78.5 Mb deletion on chromosome 6AS and a ~ 39.2 Mb telomeric deletion on 5DL in the genotype 201,353, a ~ 46.1 Mb deletion on 4BS in the genotype 201,362 or a ~ 113.4 Mb interstitial deletion on 5DL in the genotype 201,349, the A-, B- and D sub-genome chromosomes were present in all genotypes of the BC382 population (Supplementary Fig. S1). While deletions were also detected in the BC642 population, the absence of larger chromosomal fragments or even whole chromosomes was a typical phenomenon. For example, chromosomes 1A and 1AL were missing in three and one genotype, respectively, and the absence of 7B and 7BS was also detected in four and one genotype, respectively. Moreover, the absence of fragments or complete chromosomes was most often observed for chromosomes 4D (25 lines) and 5D (21 lines) in the BC642 population.

To identify introgressed regions in the BC382 and BC642 populations, the marker coverage of each BC genotype was normalised against the coverage of markers from the parental wheat (Mv9kr1) or *Ae. biuncialis* (MvGB382 or MvGB642) accessions. For chromosome regions present in a BC genotype and having the same haplotype as the parent, a coverage ratio of ~ 1 was expected, while the chromosome regions missing in the BC lines relative to the parents have a coverage value of ~ 0.

**Fig. 3:**
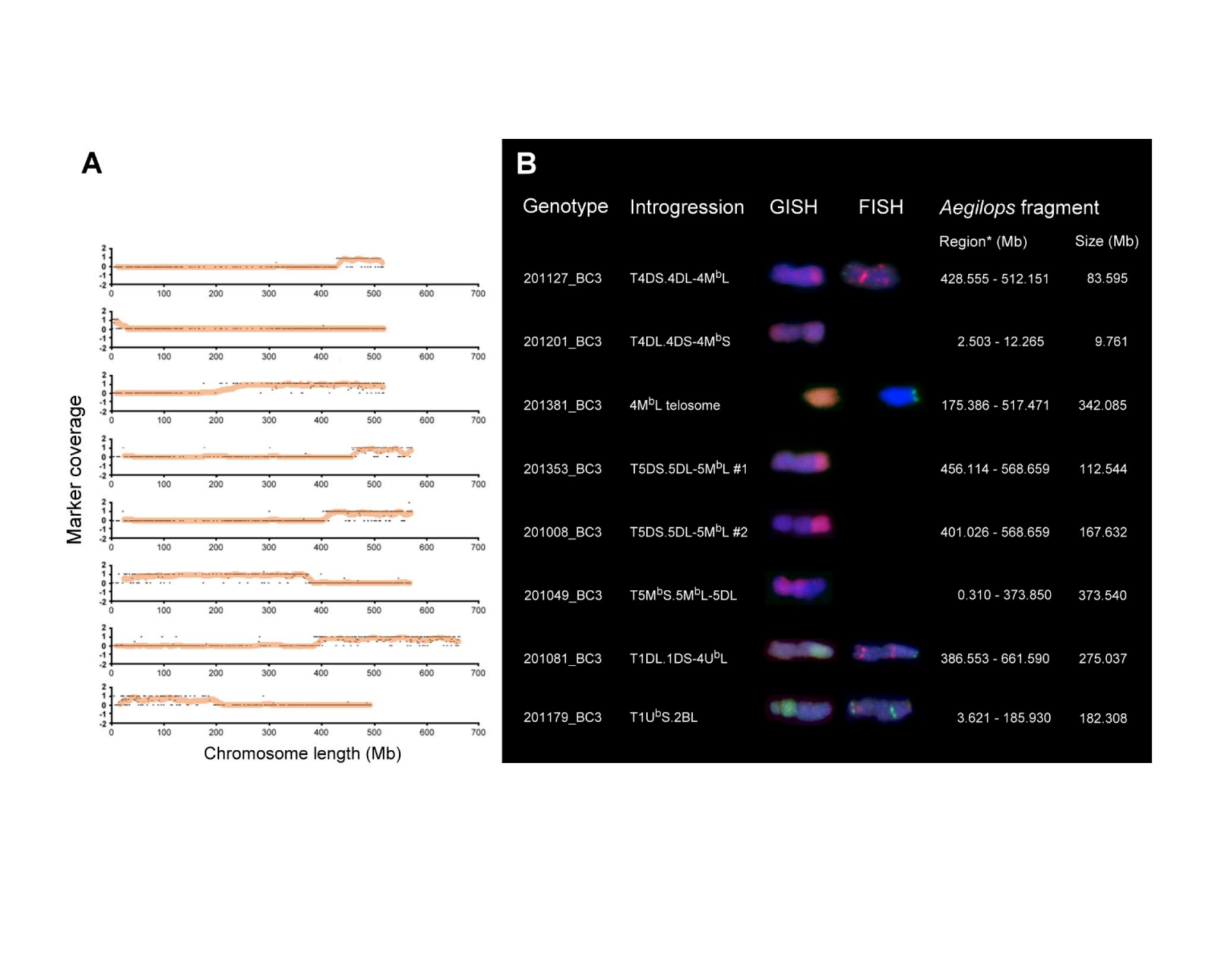
Marker coverage of the *Ae. biuncialis* chromosomes (**A**) involved in *T. aestivum*-*Ae. biuncialis* translocations identified in the BC_3_ genotypes by DArT markers (**B**). The identification of wheat-*Aegilops* translocations and the *Aegilops* telosome 4ML was validated by GISH using U- (green) and M-genomic (red) probes and by FISH using probes for Afa-family (red), pSc119.2 (green) and 45S rDNA (yellow) DNA repeats. The start- and end-positions and the size of the introgressed fragments were determined using the marker coverage data

Up to three U^b^-genome chromosome segments, including a deletion on chromosome 7U^b^ and chromosome arm 1U^b^S, and a terminal introgression of a 4U^b^L fragment, were detected in the BC_3_ populations (Fig. 1). Marker coverage and in situ hybridization using genomic- and DNA repeat probes showed that chromosome 7U^b^ has two deleted regions, a ~ 9.2 Mb terminal part of 7U^b^S and a 232.4 Mb distal part of 7U^b^L, in line 201360_BC3 (Fig. 2). FISH and GISH also confirmed the presence of the 182.3 Mb 1U^b^S arm as a 1US·2BL centric fusion in line 201179_BC3 (Fig. 3). Furthermore, cytogenetic analysis and marker data confirmed that the distal 275 Mb 4UL region is involved in aT1DL·1DS-4UL translocation detected in six BC642 lines (201077, 201079, 201081, 201082, 201087, and 201088).

Five BC_3_ lines derived from *Ae. biuncialis* accession MvGB642 (201077, 201081, 201082, 201087, and 201088) carried chromosome 5M^b^ with a 72.1 Mb interstitial deletion on the short arm (Fig. 2). Moreover, five BC642 lines (201044, 201049, 201051, 201053, and 201065) carried a 5MS·5ML-5DL translocated chromosome with a 373.5 Mb *Aegilops* segment (Fig. 3). Furthermore, a 5DS·5DL-5ML translocation (T5DS·5DL-5ML#2) was detected in four lines (201008, 201012, 201019, and 201020) with a 167.6 Mb 5M^b^L fragment (Fig. 3). Finally, another 5DS·5DL-5ML translocation (T5DS·5DL-5ML#1) was identified in the BC382 line 201,353 with a smaller (112.5 Mb) terminal segment of 5M^b^L.

Fragments of chromosome 4M^b^ were also detected in both BC_3_ populations. FISH, GISH, and DArT markers confirmed the presence of a 342 Mb 4M^b^L chromosome arm as a telosome in line 201,381 derived from the MvGB382 *Ae. biuncialis* accession (Fig. 3). In the BC642 population, a 9.7 Mb telomeric-short arm fragment was identified as a T4DL·4DS-4M^b^S translocation in line 201,201. Moreover, a terminal fragment of the long arm of 4M^b^ was also involved in a translocation T4DS·4DL-4M^b^L with 83.5 Mb of introgressed chromatin in five lines (201127, 201133, 201137, 201143, and 201146) (Fig. 3).

### Development of wheat-*Ae. biuncialis* addition lines

**Fig. 4:**
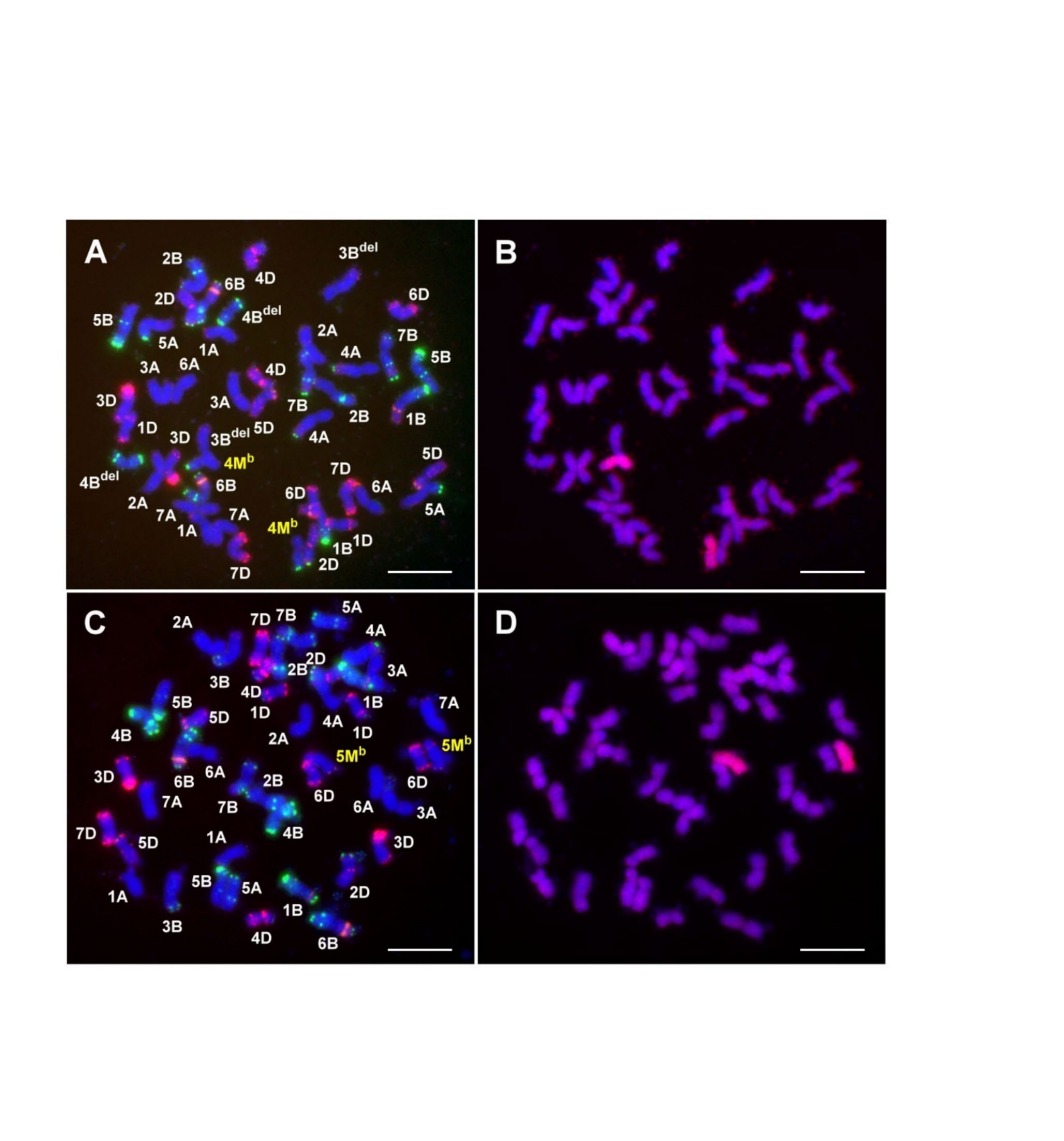
Molecular cytogenetic identification of Mv9kr1-*Ae. biuncialis* MvGB382 disomic 4M^b^ (**A, B**) and 5M^b^ (**C, D**) additions in the BC_3_F_2_ lines 232,495 and 232,490, respectively. FISH (**A, C**) on root tip mitotic metaphase chromosomes was performed with Afa-family (red), pSc119.2 (green), and pTa71 (yellow) DNA repeat probes, while GISH on the same cell (**B, D**) was performed using U- and M-genomic DNA probes visualized by green and red fluorescence, respectively. Chromosomes were counterstained by DAPI. Scale bar = 10 μm

By combining DArTseq genotyping and molecular cytogenetic approaches, we also identified four (201383, 201384, 201376, 201362) and twelve (201358, 201359, 201396, 201397, 201400, 201403, 201404, 201407, 201409, 201412, 201418, 201422) BC382 lines carrying a single whole chromosome 4M^b^ and 5M^b^, respectively, as monosomic additions. Due to unbalanced meiotic segregation, monosomic additions are not genetically stable and alien chromosomes may be eliminated from the next generation. Therefore, the BC_3_ lines were self-pollinated for two consecutive generations to produce BC_3_F_2_ lines. GISH and FISH screening identified a BC_3_F_2_ line 232,495 with 44 chromosomes carrying a pair of chromosomes 4M^b^ (Fig. 4). FISH with probes for Afa-family, pSc119.2 and 45 S rDNA confirmed that chromosomes 3B and 4B with typical karyotype (3B: telomeric and subtelomeric pSc119.2 or Afa signals on the short and long arms, respectively, 4B: strong pSc119.2 signals on the short arm telomere and at telomeric, subtelomeric and interstitial positions on the long arm) are missing, and terminal deletions were detected on the short arm of 3BS and on the long arm of 4BL (Fig. 4A).

Molecular cytogenetic analysis of the BC_3_F_2_ line 232,490 confirmed the presence of chromosome 5M^b^ in disomic form (Fig. 4CD). FISH analysis with the same set of probes used to analyse the 4M^b^ addition showed that the entire set of wheat chromosomes with normal karyotype was present in the 5 M disomic addition.

### Morphological traits

To complement the DArTseq genotyping and cytogenetic analysis, newly obtained wheat-*Ae. biuncialis* cytogenetic stocks were grown under glasshouse conditions and their spike architecture, fertility and seed morphology were evaluated (Table 1).

The 5M^b^ disomic addition line (382) had significantly higher spike length and similar number of spikelets, but lower number of seeds than Mv9kr1. The aneuploid genotypes had lower fertility than Mv9kr1, despite having a similar (5M^b^ addition) or higher number of spikelets (4M^b^ addition). However, T5DS·5DL-5M^b^L #1 (382) had longer spikes with more spikelets than Mv9kr1, resulting in a similar number of seeds per spike but lower fertility. In contrast, the remaining translocation, T5DS·5DL-5M^b^L #2, derived from the MvGB642 accession, had similar spike length and spikelet number but significantly lower number of seeds due to lower fertility compared to the wheat parent, because it was not compensated by the higher spikelet number as in the previous case. However, it should be noted, that the wild type chromosome 5M^b^ of the MvGB642 *Aegilops* accession compensated the loss of 5D in the 5M^b^(5D) substitution, including spike characteristics and fertility, as previously reported by Farkas et al. (2023).

**Fig. 5:**
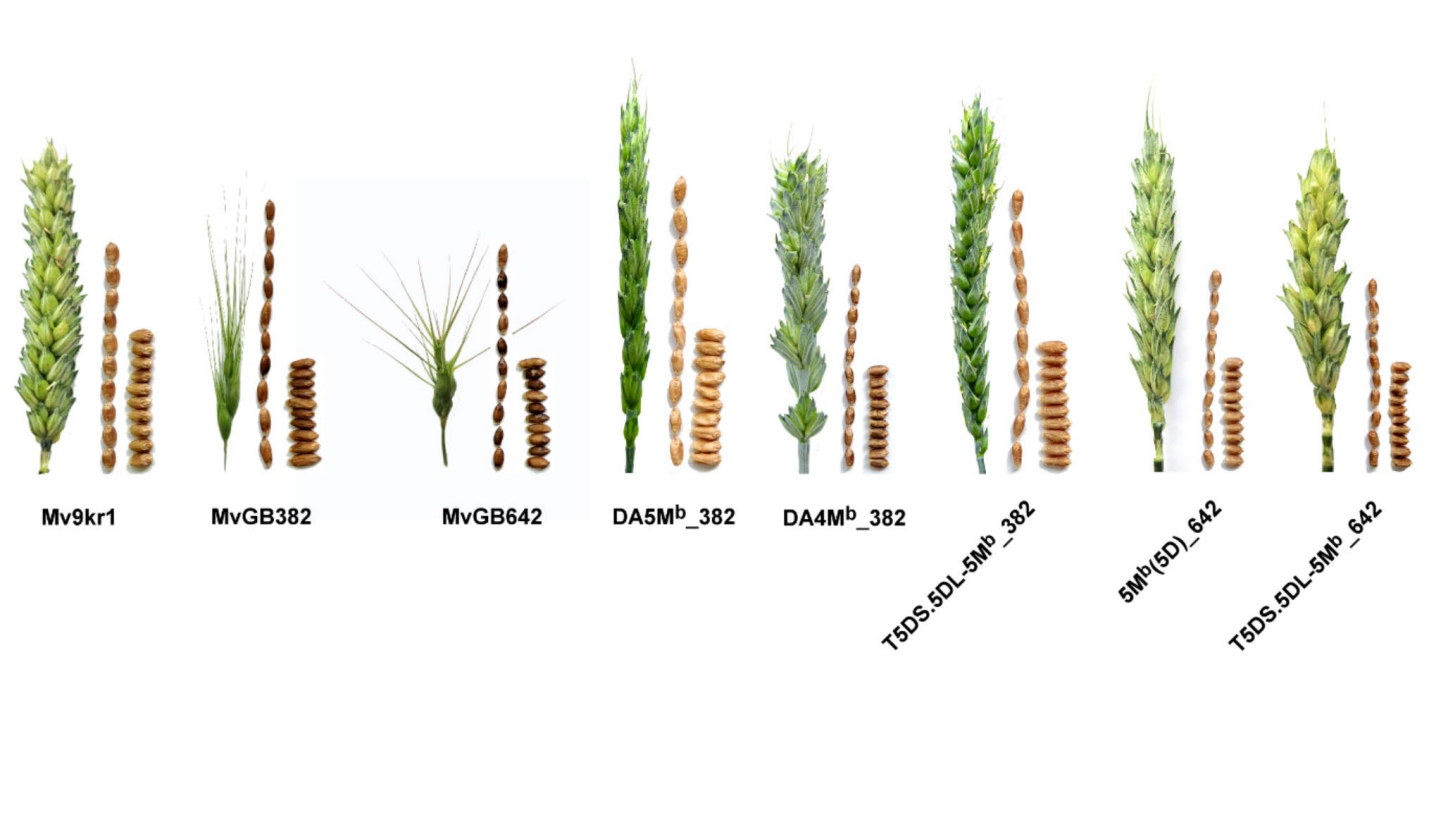
Spike and seed morphology of parental wheat (Mv9kr1) and *Ae. biuncialis* accessions (MvGB382 and MvGB642), Mv9kr1-*Ae. biuncialis* disomic additions 5M^b^ (DA5M^b^_382), 4M^b^ (DA4M^b^_382), wheat-*Ae. biuncialis* 5D/5M^b^ translocations (T5DS·5DL-5M^b^_382, T5DS·5DL-5M^b^_642) and disomic substitution 5M^b^(5D). The figure shows that the terminal 5M^b^L fragment of *Ae. biuncialis* accession MvGB382 could contribute to increased seed length even in the wheat background (Mv9kr1) as shown by the disomic 5M^b^ addition and translocation T5DS·5DL-5M^b^_382

The analysis of seed architecture (Table 2) showed that the translocation line T5DS·5DL-5M^b^L #1 (382), besides having higher fertility and more seed per spike, had a higher TKW due to longer seeds compared to the T5DS·5DL-5M^b^L #2 derived from *Ae. biuncialis* accession MvGB642. In line with this observation, the parental *Ae. biuncialis* genotypes showed similar differences in seed characteristics, as the MvGB382 accession had longer seeds and higher TKW values than those of MvGB642. We also found that the 5M^b^ addition line had longer seeds compared to the 5M^b^(5D) substitution as well as the parental wheat Mv9kr1 (Table 2; Fig. 5). This indicates significant genetic variability of important agronomic traits in the two *Ae. biuncialis* accessions that may be expressed in the wheat genetic background.

## Discussion

Efficient use of genetic diversity of *Aegilops* represented by gene bank accessions in alien introgression wheat breeding relies on the ability to track the alien chromatin in chromosome addition/translocation wheat lines. The present study investigated two BC_3_ populations derived from two *Aegilops* accessions and demonstrated that DArTseq marker technology is an effective approach for detecting alien chromatin and precisely identifying karyotypic changes in breeding lines.

In the last decade, high-density SNP genotyping platforms have become increasingly popular for detecting alien introgressions in wheat. Array-based genotyping chips with fixed SNP content represent one group of these approaches. Using an Axiom^®^ 35 K array containing a subset of SNPs from a high-density Axiom Wheat 820 K array (Winfield et al. 2016), King et al. (2017) detected 218 genome-wide wheat/*Am. muticum* introgressions. While the arrays exhibit high reproducibility and low genotyping error rates, their use is limited to the species whose sequences were used to develop them (Winfield et al. 2016) and they suffer from significant ascertainment bias in SNP detection in case of wild crop relatives (Geibel et al. 2021; Sun et al. 2020). Moreover, a relatively high cost per sample is another limitation to the widespread use of array-based genotyping platforms. SNP detection of low-cost genotyping-by-sequencing (GBS) platforms does not rely on prior sequence information and therefore represents an attractive alternative to identify non-model alien chromatin introgressed into cereal species such as barley (Wendler et al. 2014) and wheat (Keilwagen et al. 2022; Nyine et al. 2019).

In the present study, DArTseq, a GBS genotyping platform, was used to detect *Ae. biuncialis* chromatin in the wheat genetic background. The number of markers identified on the U^b^-genome chromosomes was lowest on 1U^b^ and 3U^b^ (714 and 851 markers, respectively), while we found significantly more markers on chromosomes 2U^b^, 5U^b^ and 7U^b^ (1,261, 1,291 and 1,320 markers, respectively). This phenomenon may be related to the relative size of the chromosomes. In line with this, previous flow cytometric analyses and microscopic measurements of mitotic metaphase chromosomes in the diploid progenitor *Ae. umbellulata* showed that chromosomes 1U, 3U and 6U were smaller as compared to the remaining chromosomes 2U, 4U, 5U and 7U (Molnár et al. 2011, 2016; Said et al. 2021). A similar trend was observed for the relatively small M-genome chromosomes 1M^b^, 4M^b^, and 6M^b^ and longer chromosomes 2M^b^, 3M^b^, 5M^b^ and 7M^b^ (Molnár et al. 2011, 2016; Said et al. 2021).

We also observed non-homogenous distribution of DArT markers along the chromosomes, with a general tendency for marker density to increase from the centromeric/pericentromeric regions towards the telomeric regions. This is consistent with the expectation that marker coverage is higher in gene-rich, non-methylated distal regions of chromosomes where recombination frequency is higher than in methylated proximal regions (Akhunov et al. 2003; Kalinka and Achrem 2020). Additionally, high methylation level inhibits restriction by methylation-sensitive *PstI* enzyme used for genome complexity reduction, which favours marker identification in euchromatic distal chromosomal regions. In a work parallel to the present study, using the MvGB642 x MvGB382 segregating genetic map of *Ae. biuncialis* also showed that the recombination frequency was higher in the distal regions of the U^b^ and M^b^ sub-genome chromosomes relative to the centromeric/pericentromeric regions. Nevertheless, non-homogeneous marker distribution did not prevent the detection of individual wheat and *Ae. biuncialis* chromosomes and allowed identification of intergenomic chromosomal rearrangements that could not previously be detected by PCR-based markers or in situ hybridization (Farkas et al. 2023).

Significant differences were found between the inheritance of the U^b^ and M^b^ sub-genome chromosomes in both BC_3_ populations, indicating that the U^b^ chromosomes were more often eliminated, while the chromosomes of M^b^ sub-genome were more frequently transferred to the next generation. A possible reason could be that the M^b^ genome retained significant macro collinearity with the corresponding wheat chromosomes. In contrast, the evolution of the U genome included significant genome rearrangement, as demonstrated by PCR markers and single-gene FISH probes specific for conserved orthologous genes on diploid progenitors (Molnár et al. 2013, 2016; Said et al. 2021). Similar wheat-*Aegilops* genomic relationships were further confirmed in the allotetraploid *Ae. biuncialis* by the MvGB382 x MvGB642 segregating genetic map at much higher resolution. These results agree with the findings of Edae et al. (2016) based on genetic mapping in *Ae. umbellulata*, and with the chromosome-scale reference genome sequence of *Ae. umbellulata* (Abrouk et al. 2023). In accordance with the reduced wheat-U-genome collinearity, Türkösi et al. (2022) observed reduced meiotic chromosome pairing between U^b^ and wheat chromosomes in wheat-*Ae. biuncialis* F_1_ hybrids compared to M^b^ chromosomes. Presumably, U^b^ chromosome univalents, observed more frequently in meiotic metaphase I, show unbalanced segregation, which may lead to preferential elimination of the U^b^ chromosomes from the progeny.

Another reason contributing to the preferential transfer of the M^b^ sub-genome chromosomes to the next generation, in particular chromosomes 4M^b^ and 5M^b^, could be the presence of a gametocidal gene. Preferential inheritance of certain *Aegilops* chromosomes often poses difficulties for wheat introgression breeding programs (Said et al. 2024). Gametocidal genes ensure their inheritance by selectively aborting gametes that do not carry them (Endo 1990; Endo and Gill 1996; Friebe et al. 1999; Said et al. 2024). Several *Aegilops* species have been reported to contain gametocidal genes, including *Ae. geniculata*, another allotetraploid species with the U and M genomes, on chromosome 4M^g^ (Endo 2007; Kwiatek et al. 2016, 2017; Tsujimoto and Tsunewaki 1984). A possible gametocidal effect of chromosomes 4M^b^ and 5M^b^ has also been reported for the *Ae. biuncialis* accession MvGB642 (Farkas et al. 2023). Terminal and interstitial deletions and the absence of entire chromosome arms detected in both BC_3_ populations in the present study support the gametocidal effect of chromosome 4M^b^ and 5M^b^ originating from accessions MvGB642 and MvGB382.

Using DArTseq markers, we identified *Aegilops* chromatin in several wheat-*Ae. biuncialis* MvGB642 intergenomic translocations, which was not possible using PCR-based markers (Farkas et al. 2023). Identification of translocations T4DS·4DL-4ML, T4DL·4DS-4MS, T5MS·5ML-5DL and T5DS·5DL-5ML#2 from MvGB642 and T5DS·5DL-5ML#1 from MvGB382 indicates that the terminal translocations contain M^b^ sub-genome regions homoeologous with the missing D genomic chromatin. These results indicate frequent homoeologous recombination between M^b^ sub-genome and D genome of wheat, consistent with the high frequency of M^b^-wheat meiotic chromosome pairing in wheat x *Ae. biuncialis* F_1_ hybrids (Türkösi et al. 2022). Terminal translocations 5D-5M^g^ were also detected by Tiwari et al. (2014) using 2,178 5M^g^S-specific SNPs which were developed by sequencing flow-sorted chromosome 5M^g^. Later, Koo et al. (2017) identified a homoeologous recombination-promoting factor(s) in the proximal regions of 5M^g^ that suppressed the activity of the *Ph1* locus, the major component of genetic regulation ensuring diploid-like meiosis in hexaploid wheat. The authors detected 5M^g^-5D meiotic metaphase I pairing frequency of 83.4% in cv. Chinese Spring wheat double monosomic for chromosomes 5M^g^ and 5D in the presence of active *Ph1* locus and recovered 26 (24.8%) 5M^g^-5D recombinants in the progeny. The presence of M^b^-wheat homoeologous recombination in the BC642 and BC382 populations indicates that a similar *Ph1*-suppressing genetic factor may also be present in *Ae. biuncialis*.

Our preliminary results on the wheat-*Aegilops* addition, substitution, and translocation lines suggest that the presence of *Ae. biuncialis* 5M^b^ chromatin from accession MvGB382 has a more positive effect on the seed morphology and especially on the seed length of wheat than those of 5M^b^ from MvGB642. Interestingly, the *Aegilops* parent MvGB382 also has longer seeds than the accession MvGB642 although the difference was above the 0.05 significance level (*p* = 0.052). Presumably the distal third of chromosome 5M^b^ long arm carries quantitative trait loci (QTL) affecting seed morphology, as the seed of T5DS·5DL-5ML#1 from MvGB382 was also longer than that of T5DS·5DL-5ML#2 from MvGB642. Interestingly, a recent study by Cao et al. (2020) identified a QTL strongly affecting TKW on 5DL spanning from 314.9 Mb to 557.9 Mb. Using a Doumai/Shi4185 recombinant inbred line (RIL) population Song et al. (2022) later delimited this QTL to a 409.9-413.8 Mb region on 5DL. Considering the preserved synteny between the D and M^b^ genomes, it seems logical to assume that an alternative gene variant could be found on the introgressed 5M^b^ chromosome fragment affecting seed morphology. Generation of additional 5M^b^/5D recombinants with different recombination breakpoints and a reference genome assembly for the M-genome of *Aegilops* should help to unravel the effect of 5M^b^ chromatin on wheat seed morphology.

In the present study, we developed new wheat Mv9kr1-*Ae. biuncialis* MvGB382 disomic addition lines representing chromosomes 4M^b^ and 5M^b^. The genetic map of *Ae. biuncialis* and the comparative single-gene FISH map of the M genome of *Ae. comosa* and the D genome of hexaploid wheat (Said et al. 2021) indicated that chromosomes 4M and 5M share significant homology and show collinearity with the chromosomes of the corresponding wheat homeolog groups 4 and 5. Previous studies suggested that chromosomes 5U^g^ and 5M^g^ positively affected the water-soluble pentosan content and dietary fibre composition of wheat (Rakszegi et al. 2017, 2019). The newly produced addition and introgression lines did not have high fertility, which is most likely due to subtle changes in the wheat background. It is highly probable that after a few additional backcross generations, the wheat background will be improved and the beneficial effects of the *Aegilops* chromosomes will become more apparent. Moreover, several leaf rust and stripe rust resistance genes were previously identified on group 5 chromosomes of *Aegilops*, such as *Lr57* from *Aegilops geniculata* (Kuraparthy et al. 2009), *Lr76* from *Ae. umbellulata* (Bansal et al. 2020), and *LrP* from *Ae. peregrina* (Narang et al. 2019). Since the *Ae. biuncialis* accession MvGB642 is resistant to leaf rust, the progenies of BC642 lines, frequently carrying 5M^b^ chromatin, could be promising sources of leaf rust resistance. However, additional work will be needed to map the putative leaf rust resistance gene in the Mv9kr1 background.

The analysis and use of diverse gene bank accessions is crucial in wheat pre-breeding, to identify new gene variants for adaptation to changing environments. For example, CIMMYT has produced numerous synthetic hexaploid wheats (SHWs) using different *Ae. tauschii* accessions with new morphological and agronomic traits and improved resistance to biotic and abiotic stresses (Dreisigacker et al. 2008; Rosyara et al. 2019). These examples underscore the need for similar efforts with other species, such as *Ae. biuncialis*, to make significant advances in wheat breeding.

The present study demonstrated that DArTseq effectively detects *Ae. biuncialis* chromatin and identifies karyotype changes in alien introgression lines of wheat. Although high-density SNP genotyping platforms are popular for detecting alien introgressions, their cost and species-specificity highlight the advantages of low-cost, sequence-independent platforms. DArTseq, a genotyping-by-sequencing platform of this kind was used to identify several new introgression and translocation lines. We developed new wheat Mv9kr1-*Ae. biuncialis* MvGB382 disomic addition lines representing chromosomes 4M^b^ and 5M^b^, whose spike morphology and fertility indicate their potential for developing new pre-breeding materials. The findings highlight the importance of incorporating diverse genetic resources to enhance wheat breeding efforts.

## Electronic supplementary material

Below is the link to the electronic supplementary material.

Supplementary Material 1

Supplementary Material 2

Supplementary Material 3

Supplementary Material 4

Supplementary Material 5

Supplementary Material 6

## Data Availability

The datasets generated during the current study are available as supplementary files and from the corresponding author on reasonable request.
