# Supplementary material for: DArTseq genotyping facilitates identification of Aegilops biuncialis chromatin introgressed into bread wheat Mv9kr1
Source: Plant Mol Biol. 2024 Nov 7;114(6):122. doi: 10.1007/s11103-024-01520-2 (PMC11543725; doi:10.1007/s11103-024-01520-2)

**Journal name: Plant Molecular Biology**

**DArTseq genotyping facilitate identification of *Aegilops biuncialis* chromatin introgressed into bread wheat Mv9kr1**

Eszter Gaál^1*^, András Farkas^1*^, Edina Türkösi^1^, Klaudia Kruppa^1^, Éva Szakács^1^, Kitti Szőke-Pázsi^1^, Péter Kovács^1^, Balázs Kalapos^1^, Éva Darkó^1^, Mahmoud Said^2,3^, Adam Lampar^2^, László Ivanizs^1†^, Miroslav Valárik^2^, Jaroslav Doležel^2^, István Molnár^1,2^

^1^Department of Biological Resources, Centre for Agricultural Research, Hungarian Research Network, Martonvásár 2462, Hungary

^2^Institute of Experimental Botany of the Czech Academy of Sciences, Centre of Plant Structural and Functional Genomics, Olomouc 77900, Czech Republic

^3^Field Crops Research Institute, Agricultural Research Centre, 9 Gamma Street, Giza 12619, Egypt

*These authors contributed equally to this work

^†^corresponding author: [ivanizs.laszlo@atk.hun-ren-hu](mailto:ivanizs.laszlo@atk.hun-ren-hu)

**Supplementary Figure 1.** Summary of the chromosome complement of wheat A- (*A*), B- (B) and D- (C) genome chromosomes in the Mv9kr1/*Ae. biuncialis* BC_3_ populations (BC642, BC382). The density of wheat-specific Silico-DArT markers (expressed as number of markers per Mb) in seven homologous groups was represented as heatmaps. The genotypes of wheat (*Ta*, Mv9kr1, Chinese Spring, Mv Hombár#1, Mv Hombár#2, Mv Ménrót) and *Aegilops* accessions *(Ae, Ae. umbellulata AE740/03, Ae. comosa MvGB1039, Ae. biuncialis MvGB382, Ae. biuncialis MvGB642 and Mv9kr1/Ae. biu. MvGB642 amfiploid), wheat/Aegilops* addition lines representing the chromosomes of U- (1-7U) and M-genomes (1-7M) in wheat, and the wheat*/Ae. biuncialis BC_3_ populations (BC382: 35 lines, BC642: 44 lines)* are ordered horizontally*.*


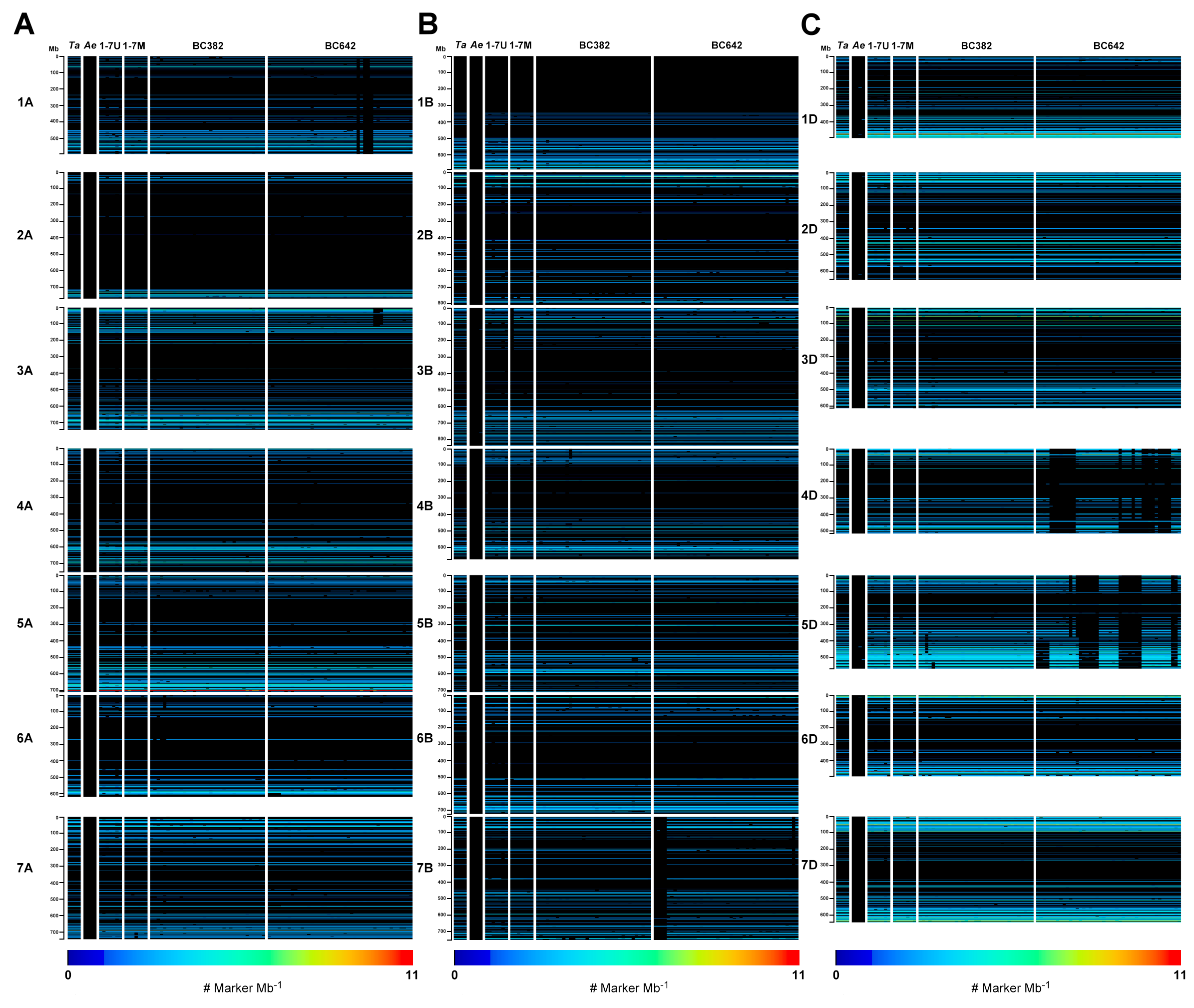

Supplement: Supplementary file 4 — Supplementary Material 4 [file 11103_2024_1520_MOESM4_ESM.docx]
