# Supplementary material for: DArTseq genotyping facilitates identification of Aegilops biuncialis chromatin introgressed into bread wheat Mv9kr1
Source: Plant Mol Biol. 2024 Nov 7;114(6):122. doi: 10.1007/s11103-024-01520-2 (PMC11543725; doi:10.1007/s11103-024-01520-2)
Supplement: Supplementary file 5 — Supplementary Material 5 [file 11103_2024_1520_MOESM5_ESM.docx]

**Journal name: Plant Molecular Biology**

**DArTseq genotyping facilitate identification of *Aegilops biuncialis* chromatin introgressed into bread wheat Mv9kr1**

Eszter Gaál^1*^, András Farkas^1*^, Edina Türkösi^1^, Klaudia Kruppa^1^, Éva Szakács^1^, Kitti Szőke-Pázsi^1^, Péter Kovács^1^, Balázs Kalapos^1^, Éva Darkó^1^, Mahmoud Said^2,3^, Adam Lampar^2^, László Ivanizs^1†^, Miroslav Valárik^2^, Jaroslav Doležel^2^, István Molnár^1,2^

^1^Department of Biological Resources, Centre for Agricultural Research, Hungarian Research Network, Martonvásár 2462, Hungary

^2^Institute of Experimental Botany of the Czech Academy of Sciences, Centre of Plant Structural and Functional Genomics, Olomouc 77900, Czech Republic

^3^Field Crops Research Institute, Agricultural Research Centre, 9 Gamma Street, Giza 12619, Egypt

*These authors contributed equally to this work

^†^corresponding author: [ivanizs.laszlo@atk.hun-ren-hu](mailto:ivanizs.laszlo@atk.hun-ren-hu)

**Supplementary Table 1.**

The plant material used in the present study.

|  |  | Investigated by |  |
| --- | --- | --- | --- |
|  | Genotype | DArTseq | GISH/FISH |
| hexaploid wheat parent | Mv9kr1 | + | - |
| hexaploid wheat control | Chinese Spring (CS) | + | - |
|  | Mv Hombár#1 | + | - |
|  | Mv Hombár#2 | + | - |
|  | Ménrót | + | - |
| *Aegilops* parents | *Ae. biuncialis* MvGB382 | + | - |
|  | *Ae. biuncialis* MvGB642 | + | - |
| U genome progenitor | *Ae. umbellulata* AE740/03 | + | - |
| M genome progenitor | *Ae. comosa* MvGB1039 | + | - |
| wheat- *Ae. biuncialis* amphiploid | Mv9kr1 – *Ae. biuncialis* MvGB642 | + | - |
| wheat-*Aegilops* additions | CS-*Ae. geniculata*_1M | + | - |
|  | CS-*Ae. geniculata*_2M | + | - |
|  | CS-*Ae. geniculata*_3M | + | - |
|  | CS-*Ae. geniculata*_4M | + | - |
|  | CS-*Ae. geniculata*_5M | + | - |
|  | CS-*Ae. geniculata*_6M | + | - |
|  | CS-*Ae. geniculata*_7M | + | - |
|  | CS- *Ae. umbellulata*_1U | + | - |
|  | CS- *Ae. umbellulata*_2U | + | - |
|  | Mv9kr1- *Ae. biuncialis*_3U* | + | - |
|  | CS- *Ae. umbellulata*_4U | + | - |
|  | CS- *Ae. umbellulata*_5U | + | - |
|  | CS- *Ae. umbellulata*_6U | + | - |
|  | CS- *Ae. umbellulata*_7U | + | - |
| Mv9kr1-*Ae. biuncialis* MvGB382 BC_3_ population | 201345-201422_BC382 (35 genotypes see Supplementary Data S1-S3) | + | + |
| Mv9kr1-*Ae. biuncialis* MvGB642 BC_3_ population | 201008-201201_BC642  (44 genotypes) | + | + |
| Mv9kr1-*Ae. biuncialis* MvGB382 BC_3_F_2_ population | 232495, 232490 | - | + |
