# Supplementary material for: DArTseq genotyping facilitates identification of Aegilops biuncialis chromatin introgressed into bread wheat Mv9kr1
Source: Plant Mol Biol. 2024 Nov 7;114(6):122. doi: 10.1007/s11103-024-01520-2 (PMC11543725; doi:10.1007/s11103-024-01520-2)
Supplement: Supplementary file 6 — Supplementary Material 6 [file 11103_2024_1520_MOESM6_ESM.docx]

**Journal name: Plant Molecular Biology**

**DArTseq genotyping facilitate identification of *Aegilops biuncialis* chromatin introgressed into bread wheat Mv9kr1**

Eszter Gaál^1*^, András Farkas^1*^, Edina Türkösi^1^, Klaudia Kruppa^1^, Éva Szakács^1^, Kitti Szőke-Pázsi^1^, Péter Kovács^1^, Balázs Kalapos^1^, Éva Darkó^1^, Mahmoud Said^2,3^, Adam Lampar^2^, László Ivanizs^1†^, Miroslav Valárik^2^, Jaroslav Doležel^2^, István Molnár^1,2^

^1^Department of Biological Resources, Centre for Agricultural Research, Hungarian Research Network, Martonvásár 2462, Hungary

^2^Institute of Experimental Botany of the Czech Academy of Sciences, Centre of Plant Structural and Functional Genomics, Olomouc 77900, Czech Republic ^3^Field Crops Research Institute, Agricultural Research Centre, 9 Gamma Street, Giza 12619, Egypt

*These authors contributed equally to this work

^†^corresponding author: [ivanizs.laszlo@atk.hun-ren-hu](mailto:ivanizs.laszlo@atk.hun-ren-hu)

**Supplementary Table 2A.**

The identified *Ae. biuncialis* introgressions in hexaploid Mv9kr1 x *Ae. biuncialis* MvGB382 BC3 population (A) and Mv9kr1 x *Ae. biuncialis* MvGB642 BC3 (B)

| **BC382 population** | **Whole chromosomes** | | **Translocation/telosomes/deletions** | **Size of introgressed chromatin (Mb)** |
| --- | --- | --- | --- | --- |
|  | **M^b^** | **U^b^** |  |  |
| **201345** | **3M** |  |  |  |
| **201347** | **3M, 4M, 5M** |  |  |  |
| **201349** | **3M, 5M** |  |  |  |
| **201351** | **3M, 5M** |  |  |  |
| **201353** |  |  | **5ML** | **112,5** |
| **201354** | **3M, 5M** |  |  |  |
| **201357** | **5M** |  | **4MS** | **152,5** |
| **201358** | **5M** |  |  |  |
| **201359** | **5M** |  |  |  |
| **201360** | **4M, 5M** |  | **7US** | **337** |
| **201362** | **4M** |  |  |  |
| **201366** | **3M, 4M** |  |  |  |
| **201368** | **3M, 5M** |  |  |  |
| **201370** | **4M, 5M** |  |  |  |
| **201373** | **3M, 4M** | **2U** |  |  |
| **201375** | **3M, 4M** |  |  |  |
| **201376** | **4M, 6M** |  |  |  |
| **201378** |  |  |  |  |
| **201380** | **4M** |  |  |  |
| **201381** | **6M** |  | **4ML** | **342,1** |
| **201383** | **4M** |  |  |  |
| **201384** | **4M, 6M** |  |  |  |
| **201386** |  |  |  |  |
| **201389** |  |  |  |  |
| **201392** |  |  |  |  |
| **201396** | **5M** |  |  |  |
| **201397** | **5M** |  |  |  |
| **201400** | **5M** |  |  |  |
| **201403** | **5M** |  |  |  |
| **201404** | **5M** |  |  |  |
| **201407** | **5M** |  |  |  |
| **201409** | **5M** |  |  |  |
| **201412** | **5M** |  |  |  |
| **201418** | **5M** |  |  |  |
| **201422** | **5M** |  |  |  |

**Supplementary Table 2B.**

| **BC642 population** | **Whole chromosomes** | | **Translocation/telosomes/deletions** | **Size of introgressed chromatin (Mb)** |
| --- | --- | --- | --- | --- |
|  | **M^b^** | **U^b^** |  |  |
| **201008** |  |  | **5ML** | **167,6** |
| **201012** |  |  | **5ML** | **167,6** |
| **201019** |  |  | **5ML** | **167,6** |
| **201020** |  |  | **5ML** | **167,6** |
| **201027** | **4M** |  |  |  |
| **201028** | **4M** |  |  |  |
| **201044** | **4M** |  | **5MS** | **373,9** |
| **201045** | **4M** |  |  |  |
| **201049** | **4M** |  | **5MS** | **373,9** |
| **201051** | **4M** |  | **5MS** | **373,9** |
| **201053** | **4M** |  | **5MS** | **373,9** |
| **201061** | **4M** |  |  |  |
| **201065** | **4M** |  | **5MS** | **373,9** |
| **201077** |  |  | **5Mdel, 4UL** | **213,8-761,3, 275,0** |
| **201079** |  |  | **4UL** | **275,0** |
| **201081** |  |  | **5Mdel, 4UL** | **213,8-761,3; 275,0** |
| **201082** |  |  | **5Mdel, 4UL** | **213,8-761,3; 275,0** |
| **201087** |  |  | **5Mdel, 4UL** | **213,8-761,3; 275,0** |
| **201088** |  |  | **5Mdel, 4UL** | **213,8-761,3; 275,0** |
| **201096** |  |  |  |  |
| **201097** |  |  |  |  |
| **201106** |  | **4U** |  |  |
| **201107** |  | **4U** |  |  |
| **201114** |  |  |  |  |
| **201118** |  |  |  |  |
| **201122** | **4M, 5M** |  |  |  |
| **201127** | **5M** |  | **4ML** | **889,2** |
| **201133** | **5M** |  | **1MS, 3MS, 4ML** | **188,7; 247,3; 88,9** |
| **201137** | **5M** |  | **4ML** | **88,9** |
| **201139** | **4M, 5M** |  | **1MS, 3MS** | **188,7; 247,3** |
| **201143** | **5M** |  | **4ML** | **88,9** |
| **201146** | **5M** |  | **4ML** | **88,9** |
| **201151** | **4M** |  | **1MS, 3MS** | **188,7; 247,3** |
| **201152** | **4M** |  | **1MS, 3MS** | **188,7; 247,3** |
| **201154** | **4M** |  | **1MS, 3MS** | **188,7; 247,3** |
| **201164** | **4M** |  | **1MS, 3MS** | **188,7; 247,3** |
| **201170** | **4M** |  | **1MS, 3MS** | **188,7; 247,3** |
| **201171** | **4M** |  | **1MS, 3MS** | **188,7; 247,3** |
| **201174** | **4M** |  |  |  |
| **201179** | **4M** |  | **1US** | **185,9** |
| **201182** | **4M** |  |  |  |
| **201195** |  |  | **5Mdel** | **213,7-761,3** |
| **201196** |  |  | **5Mdel** | **213,7-761,3** |
| **201201** |  |  | **4MS** | **122,7** |
